# Real-world association between systemic corticosteroid exposure and complications in US patients with severe asthma

**DOI:** 10.1186/s13223-024-00882-y

**Published:** 2024-03-26

**Authors:** Thomas B Casale, Thomas Corbridge, Guillaume Germain, François Laliberté, Sean D MacKnight, Julien Boudreau, Mei S Duh, Arijita Deb

**Affiliations:** 1 Division of Allergy/Immunology, University of South Florida, Tampa, FL USA; 2 US Medical Affairs, GSK, Durham, NC USA; 3 Health Economics and Outcomes Research, Groupe d’analyse, Montréal, QC Canada; 4 Health Economics and Outcomes Research, Analysis Group Inc, Boston, MA USA; 5 Value Evidence and Outcomes, GSK, Upper Providence, PA USA

**Keywords:** Asthma, Systemic corticosteroid, Systemic corticosteroid-related complication, Gastrointestinal, Cardiovascular, Metabolic, Endocrine, Central nervous system, Ophthalmologic

## Abstract

**Background:**

Systemic corticosteroid (SCS) use remains widespread among patients with severe asthma, despite associated complications.

**Objective:**

Evaluate the association between cumulative SCS exposure and SCS-related complications in severe asthma.

**Methods:**

This retrospective, longitudinal study used claims data from the Optum Clinformatics Data Mart database (GSK ID: 214469). Eligible patients (≥ 12 years old) had an asthma diagnosis and were divided into two cohorts: SCS use and non/burst-SCS use. Patients in the SCS use cohort had a claim for a daily prednisone-equivalent dose ≥ 5 mg SCS following ≥ 6 months of continuous SCS use; those in the non/burst-SCS cohort had no evidence of continuous SCS use and had a non-SCS controller/rescue medication initiation claim. For each cohort, the date of the qualifying claim was the index date. SCS users were further stratified by SCS use during each quarter of follow-up: low (≤ 6 mg/day), medium (> 6–12 mg/day), high (> 12 mg/day), and continuous high (≥ 20 mg/day for 90 days). SCS-related complications were evaluated in the quarter following SCS exposure. The adjusted odds ratios (OR) of experiencing SCS-related complications during follow-up in each of the SCS use groups versus the non/burst SCS cohort were calculated using generalized estimating equations models.

**Results:**

SCS and non/burst-SCS use cohorts included 7473 and 89,281 patients (mean follow-up: 24.6 and 24.2 months), respectively. Compared with the non/burst-SCS use cohort, medium, high, and continuous high SCS use was associated with greater odds of any SCS-related complication (adjusted OR [95% confidence interval]: 1.30 [1.21, 1.39], 1.49 [1.35, 1.64] and 1.63 [1.40, 1.89], respectively) including increased acute gastrointestinal, cardiovascular, and immune system-related complications, and chronic cardiovascular, metabolic/endocrine, central nervous system, bone-/muscle-related, ophthalmologic, and hematologic/oncologic complications. Low-dose SCS use was also associated with significantly increased odds of acute gastrointestinal and immune system-related complications, and chronic bone-/muscle-related and hematologic/oncologic complications versus the non/burst-SCS use cohort.

**Conclusion:**

SCS use, even at low doses, is associated with increased risk of SCS-related complications among patients with severe asthma.

**Supplementary Information:**

The online version contains supplementary material available at 10.1186/s13223-024-00882-y.

## Background

Patients with severe asthma, accounting for 3–10% of the total asthma population, require treatment with high-dose inhaled corticosteroids (ICS) plus a second controller and/or SCS to control symptoms [1, 2]. However, their asthma can remain uncontrolled despite these therapies [1, 2]. Historically, a primary component of severe asthma management has been maintenance treatment with and/or rescue bursts of SCS, which includes both oral corticosteroids (OCS) and parenteral corticosteroids delivered via subcutaneous and intravenous routes [2–4]. However, the adverse effects of short- and long-term SCS use are well documented and include osteoporosis, bone fractures, cardiovascular disease, gastrointestinal conditions, impaired immune response, alterations in glucose and lipid metabolism, and psychiatric disturbances [5–10]. Importantly, while the risk of SCS-related complications has been shown to increase in a dose-dependent manner [6], even patients with low SCS exposure are at greater risk of SCS-related complications than non-users [8]. In addition to the substantial clinical burden associated with SCS-related complications, the increased risk also translates to a greater economic burden in patients with severe asthma, with higher annual healthcare costs and healthcare resource utilization (HCRU) in SCS users versus non-users [5, 6, 8, 9]. 

The Global Initiative for Asthma (GINA) guideline (2023) acknowledges the substantial adverse effects of OCS use and recommends only considering maintenance OCS as a last resort [2]. Several biologic agents have been approved for the treatment of severe asthma, including omalizumab, reslizumab, tezepelumab, mepolizumab, benralizumab, and dupilumab [11–22]. These agents have demonstrated OCS-sparing effects both in clinical trials and in real-world settings [16, 19, 20, 22–24]. While the cost effectiveness of biologics has been debated [25], and reductions in exacerbations requiring hospitalization are the primary driver of cost effectiveness with biologic therapy [26, 27], chronic OCS reduction has also been reported to contribute to the lifetime discounted cost effectiveness of biologic therapy [28]. Despite the introduction of biologics in several countries worldwide, evidence suggests that patients remain unnecessarily reliant on OCS for the management of asthma [29]. Currently, there are limited data available on the association between cumulative SCS exposure and related complications since the introduction of biologic therapies; therefore, updated assessments are needed.

The objective of this real-world, retrospective study was to evaluate the association between cumulative SCS exposure and SCS-related complications and HCRU in patients with severe asthma, stratified by SCS use.

## Methods

### Data source

This was a retrospective, longitudinal study using medical, and pharmacy claims data from the Optum Clinformatics Data Mart (CDM) database (GSK ID: 214469). Optum CDM covers approximately 15–20 million annual lives of UnitedHealth Group members in all US census regions and includes data from January 1, 2014, to December 31, 2020. The database comprises both commercial and Medicare Advantage health plan data. This study used fully de-identified data compliant with the Health Insurance Portability and Accountability Act; therefore, informed consent, ethics committee or institutional review board approval was not required.

### Study design

**Fig. 1:**
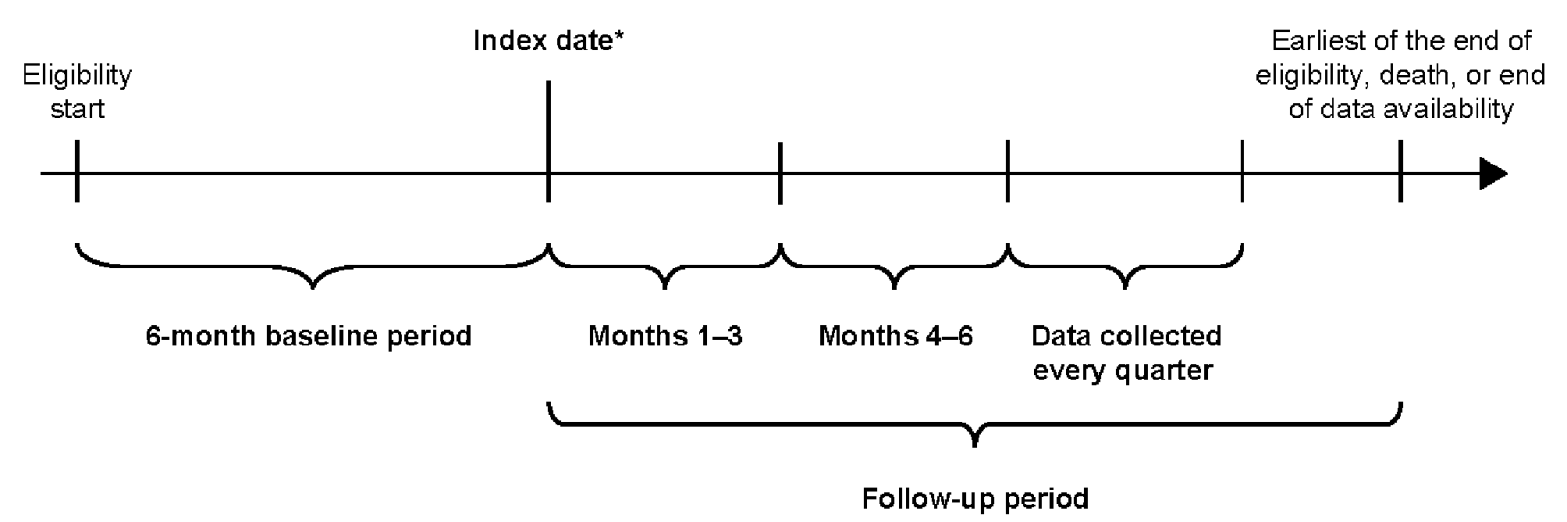
Study design. *First pharmacy/medical claim date for SCS ≥ 5 mg/day following 6 months of continuous (≥ 5 mg/day for 6 months without any gap > 14 days between claims) SCS use (SCS use cohort) or date of initiation of any non-SCS asthma controller/rescue medication (non/burst-SCS use cohort). SCS, systemic corticosteroid

The study design is outlined in Fig. 1. Two cohorts were defined: the SCS use and non/burst-SCS use cohorts (see **Patients** section for eligibility criteria of each cohort). The index date for patients in the SCS use cohort was the date of the first pharmacy or medical claim for SCS with a daily prednisone-equivalent dose ≥ 5 mg following 6 months of continuous SCS use (defined as ≥ 5 mg/day of SCS for 6 months without any gap of > 14 days between the last day of supply of a claim and the next claim; Fig. 1).

The 6-month period of continuous SCS use, and the ≥ 5 mg daily prednisone equivalent threshold were used for consistency with prior retrospective studies and a randomized clinical trial [6–8, 16]. The index date for patients in the non/burst-SCS use cohort was defined as the date of initiation of any asthma controller or rescue medication other than SCS (Fig. 1); if more than one asthma medication other than SCS was taken among patients in the non/burst-SCS cohort, the index date was randomly assigned among the asthma medication initiation dates. The index date for the non/burst-SCS cohort was selected randomly among asthma medication initiation dates instead of selecting the first asthma initiation date to avoid indexing non/burst-SCS patients at a systematically earlier timepoint in their treatment journey relative to the SCS cohort.

Data were collected for the 6-month period preceding the index date (baseline period; for evaluation of demographic and clinical characteristics) and for a variable-length, follow-up period. The length of the follow-up period spanned from the index date up to the end of eligibility, death, or end of data availability, whichever occurred first.

To account for the change in cumulative SCS exposure over time, the follow-up period was segmented into quarterly (90-day) intervals, and only complete quarters of follow-up were assessed. The association between cumulative SCS exposure (measured as the average daily dose received from index up to the first day of each quarter) and study outcomes was evaluated repeatedly for each quarter of follow-up. The first quarter post-index was reserved for the assessment of SCS exposure, whereas outcomes were evaluated starting in the second quarter post-index.

### Patients

Eligible patients were ≥ 12 years of age on the index date, had ≥ 6 months of continuous eligibility both before and after the index date and had ≥ 2 primary or secondary asthma diagnosis codes any time before or on the index date. International Classification of Diseases (ICD)-9/10-clinical modification (CM) diagnosis codes are included in Additional File 1 (Supplementary Table 1). Patients with severe asthma included in the SCS use cohort were required to have ≥ 6 months of continuous use of oral or parenteral SCS (see Study design section for definition of continuous use) with a daily dose equivalent to ≥ 5 mg prednisone, and ≥ 1 dispensing or administration of SCS with a daily dose equivalent to ≥ 5 mg prednisone after the previous 6 months of continuous SCS use. The non/burst-SCS use cohort included patients with asthma and no claim for any OCS or parenteral SCS used to treat asthma at any time during their eligibility period, except for those received as part of an outpatient (OP) or emergency room (ER) exacerbation, and with ≥ 1 dispensing for a controller or rescue medication for asthma other than SCS. OP/ER exacerbations were defined as an asthma-related OP or ER visit with ≥ 1 claim for an SCS within − 4/+5 days of the encounter. Asthma-related visits were identified as those with a claim with a primary diagnosis of asthma.

Patients from both cohorts were excluded if they had ≥ 1 primary or secondary diagnosis of cancer of the respiratory and intrathoracic system any time before or on the index date, or if they had ≥ 1 primary or secondary diagnosis for other conditions where OCS is commonly used, including rheumatoid arthritis, Crohn’s disease, ulcerative colitis, systemic lupus erythematosus, or multiple sclerosis, at any time during the study period.

### Outcomes

The primary outcome was the frequency of SCS-related complications (any, acute, and chronic) during the follow-up period, identified based on the presence of associated diagnosis codes (list of all SCS-related complications defined in Additional File 1; Supplementary Table 2). Secondary outcomes included HCRU rates due to SCS-related complications during the follow-up period, and SCS treatment patterns during the follow-up period (SCS use cohort only). HCRU due to SCS-related complications included IP visits (hospitalizations and skilled nursing facilities), ER visits, OP visits, and other visits (including home services and hospice visits), and were identified with a primary or secondary diagnosis code for any SCS-related complication. Patient demographics and characteristics at baseline were also described.

For the assessment of SCS-related complications and HCRU due to SCS-related complications, patients in the SCS use cohort were stratified into mutually exclusive subgroups based on SCS exposure, measured as the average daily dose (prednisone-equivalent) from the index date up to the first day of each measurement quarter using an open-cohort approach. Further, patients were classified into low (≤ 6 mg/day), medium (> 6–12 mg/day), and high (> 12 mg/day) SCS exposure subgroups. In addition, patients using ≥ 20 mg/day of SCS for 90 days (assessed in the 90 days preceding each quarter) were included in a non-mutually exclusive continuous high-dose subgroup [30–32]. Finally, patients in the SCS use cohort with ≥ 3 SCS bursts or ≥ 4 SCS bursts between the index date and the first day of each quarter were included in two further non-mutually exclusive subgroups. An SCS burst was defined as a pharmacy or medical claim for SCS with 2–28 days of supply and an average daily dose equivalent to prednisone ≥ 20 mg. Multiple SCS bursts fewer than 14 days apart were considered as a single SCS burst.

### Statistical analysis

Patient baseline demographics and characteristics were assessed descriptively, with mean, standard deviation (SD), and median values for the continuous variables, and frequencies and proportions for the categorical variables. Differences between the SCS use and non/burst-SCS use cohorts were assessed using standardized differences, with a difference between cohorts ≥ 10% considered important [33]. 

The frequency of SCS-related complications and the rates of HCRU due to SCS-related complications during the follow-up period were analyzed using adjusted odds ratios (OR) with 95% confidence intervals (CI) calculated from generalized estimating equations (GEE) models using a binomial distribution for complications, and a Poisson distribution for HCRU. Adjusted GEE models controlled for the following baseline covariates: age, sex, year of index date, region, insurance plan type, physician specialty, Quan-Charlson comorbidity index (CCI) score [34], respiratory and other medications (≥ 5% prevalence in either cohort), asthma-related exacerbations (IP and OP/ER) during baseline and on the index date, all-cause and asthma-related HCRU and costs (IP, ER, OP, and other visit components), Elixhauser and asthma-related comorbidities (≥ 5% prevalence in either cohort), and baseline SCS-related complications (≥ 5% prevalence in either cohort).

SCS treatment patterns were assessed per quarter and were also reported over the entire follow-up period. Metrics evaluated over the entire follow-up period were reported per patient per quarter and were weighted by each patient’s length of observation (in complete quarters). A sensitivity analysis was conducted comparing SCS complications among the low-dose SCS use subgroup versus the non/burst-SCS use cohort after removing patients with any SCS exposure during follow-up from the non/burst-SCS user cohort (i.e., patients with SCS exposure as part of an OP or ER exacerbation during the follow-up).

## Results

### Patient population

Overall, 7473 patients in the SCS use cohort and 89,281 in the non/burst-SCS use cohort met the eligibility criteria and were included in the analysis (Additional File 2; Supplementary Fig. 1).

Compared with the non/burst-SCS use cohort, the SCS use cohort was older, had a greater proportion of Medicare enrollees, and had a lower proportion of patients managed by primary care physicians at index date (Table 1).

**Table 1:** Baseline demographics at index

Demographics	SCS use cohort *N* = 7473	Non/burst-SCS use cohort *N* = 89,281	Standardized difference (%)
Observation period, months, mean (SD) [median]	24.6 (14.9) [21]	24.2 (15.1) [20]	2.4
Age, years, mean (SD) [median]	67.4 (13.6) [69]	52.2 (20.6) [55]	86.8*
Female, n (%)	4682 (63)	59,249 (66)	7.8
Region, n (%)			
South	3246 (43.4)	37,373 (41.9)	3.2
West	1664 (22.3)	21,869 (24.5)	5.3
Midwest	1514 (20.3)	18,700 (20.9)	1.7
Northeast	1046 (14.0)	11,225 (12.6)	4.2
Unknown	3 (0.0)	114 (0.1)	3.0
Insurance plan type, n (%)			
Medicare	5877 (78.6)	37,283 (41.8)	75.4*
Commercial	1596 (21.4)	51,998 (58.2)	75.4*
Physician specialty^†^, n (%)			
Primary care	3502 (46.9)	52,432 (58.7)	23.8*
Respiratory specialist	1314 (17.6)	18,193 (20.4)	7.1
Pulmonologist	1070 (14.3)	10,409 (11.7)	7.9
Allergist	247 (3.3)	7839 (8.8)	23.0*

*Standardized difference ≥ 10%; ^†^based on the latest medical claim ≤ 30 days pre-index (inclusive). Includes generalist, internal medicine, pediatrician, and nurse practitioner. Allergist and pulmonologist are not mutually exclusive as patients may visit both specialists on the same day. SCS, systemic corticosteroid

During the 6-month baseline period, the SCS use cohort also had a higher mean Quan-CCI score, and more frequent medication use (including controllers and rescue medications) compared with the non/burst-SCS use cohort (Table 2). In general, all-cause, and asthma-related HCRU was higher in the SCS use cohort compared with the non/burst-SCS use cohort, with more frequent all-cause and asthma-related IP and OP visits (Table 2). The associated all-cause and asthma-related healthcare costs followed a similar trend (Additional File 1; Supplementary Table 3). In the SCS use cohort, the mean numbers of IP exacerbations and OP/ER asthma exacerbations during baseline were higher than in the non/burst-SCS use cohort (Table 2).

**Table 2:** Clinical characteristics, complications and HCRU during the 6-month baseline period

Characteristics	SCS use cohort *N* = 7473	Non/burst-SCS use cohort *N* = 89,281	Standardized difference (%)
Quan-CCI, mean (SD) [median]	2.40 (1.93) [2]	1.30 (1.28) [1]	66.7*
Controller medications, n (%)			
Any use	5267 (70.5)	32,264 (36.1)	68.8*
Inhaled corticosteroid (ICS/long-acting β_2_-agonist (LABA)	3176 (42.5)	15,190 (17.0)	55.7*
Leukotriene modifiers	2971 (39.8)	15,477 (17.3)	49.6*
Long-acting muscarinic antagonist (LAMA)	1177 (15.8)	1667 (1.9)	49.0*
Inhaled corticosteroids	1055 (14.1)	7414 (8.3)	18.4*
Multiple-inhaler triple therapy†	983 (13.2)	914 (1.0)	47.3*
Biologics	416 (5.6)	861 (1.0)	25.9*
Methylxanthines	375 (5.0)	357 (0.4)	28.4*
LAMA/LABA	211 (2.8)	290 (0.3)	20.1*
LABA	194 (2.6)	245 (0.3)	19.5*
Single-inhaler triple therapy	138 (1.8)	247 (0.3)	15.3*
Mast cell stabilizers	12 (0.2)	18 (0.0)	4.7
Rescue medications, n (%)			
Any use	6230 (83.4)	55,610 (62.3)	47.4*
Antibiotics	4946 (66.2)	41,774 (46.8)	39.1*
Short-acting β_2_-agonist (SABA)	3984 (53.3)	32,581 (36.5)	33.8*
SABA/short-acting muscarinic antagonist (SAMA)	1569 (21.0)	3344 (3.7)	52.4*
SAMA	372 (5.0)	798 (0.9)	24.2*
Healthcare resource utilization, mean (SD) [median]			
All-cause			
Inpatient visits	0.42 (0.88) [0]	0.14 (0.47) [0]	39.4*
Emergency room visits	0.91 (2.39) [0]	0.68 (1.82) [0]	11.0*
Outpatient visits	15.61 (13.25) [12]	8.20 (9.13) [6]	65.1*
Outpatient hospital visits	5.28 (9.83) [2]	1.73 (4.81) [0]	45.9*
Office visits	9.32 (7.98) [7]	6.04 (6.53) [4]	45.0*
Other outpatient visits	1.00 (4.27) [0]	0.43 (2.92) [0]	15.6*
Other visits	5.22 (10.30) [1]	1.21 (4.50) [0]	50.4*
Asthma-related			
Inpatient visits	0.06 (0.29) [0]	0.03 (0.19) [0]	10.7*
Emergency room visits	0.10 (0.73) [0]	0.12 (0.44) [0]	3.1
Outpatient visits	1.02 (2.40) [0]	0.71 (1.41) [0]	15.9*
Outpatient hospital visits	0.26 (1.28) [0]	0.13 (0.73) [0]	12.5*
Office visits	0.75 (1.88) [0]	0.57 (1.17) [0]	11.1*
Other outpatient visits	0.02 (0.16) [0]	0.01 (0.09) [0]	7.5
Other visits	0.32 (1.48) [0]	0.10 (0.75) [0]	18.6*
Exacerbations during baseline, mean (SD) [median]			
Inpatient exacerbations^‡^	0.05 (0.26) [0]	0.03 (0.19) [0]	10.0*
Outpatient/emergency room exacerbations^§^	0.40 (0.88) [0]	0.31 (0.57) [0]	11.8*
Patients with ≥ 1 exacerbation during baseline, n (%)			
Inpatient exacerbations^‡^	347 (4.6)	2603 (2.9)	9.1
Outpatient/emergency room exacerbations^§^	1760 (23.6)	23,907 (26.8)	7.4
Patients with ≥ 1 exacerbation on the index date, n (%)			
Inpatient exacerbations^‡^	13 (0.2)	76 (0.1)	2.5
Outpatient/emergency room exacerbations^§^	282 (3.8)	10,159 (11.4)	28.7*
Any SCS-related complication, n (%)	6960 (93.1)	66,805 (74.8)	49.9*
Acute			
Immune system related	4094 (54.8)	33,759 (37.8)	34.0*
Gastrointestinal	1218 (16.3)	8704 (9.7)	19.5*
Cardiovascular	480 (6.4)	2016 (2.3)	20.4*
Bone and muscle related	27 (0.4)	340 (0.4)	0.3
Chronic			
Cardiovascular	5422 (72.6)	37,886 (42.4)	60.9*
Metabolic and endocrine	5038 (67.4)	41,057 (46.0)	43.2*
Cushing’s syndrome	75 (1.0)	40 (0.0)	13.3*
Central nervous system	3865 (51.7)	31,519 (35.3)	33.1*
Bone and muscle related	2968 (39.7)	18,426 (20.6)	41.6*
Ophthalmologic	1048 (14.0)	6326 (7.1)	22.6*
Hematologic/oncologic	746 (10.0)	2930 (3.3)	26.9*
Dermatologic	131 (1.8)	2165 (2.4)	4.7
Gastrointestinal	1 (0.0)	2 (0.0)	1.3

*Standardized difference ≥ 10%; ^†^≥1 overlapping ICS, LABA and LAMA supply days; ^‡^requiring an asthma-related IP visit; ^§^requiring an asthma-related OP/ER visit with ≥ 1 SCS claim within − 4/+5 days of the encounter. Quan-CCI, Quan-Charlson comorbidity index; SCS, systemic corticosteroids

Baseline chronic SCS-related complications, including metabolic and endocrine complications, cardiovascular complications, and bone- and muscle-related complications, were more frequent in the SCS use versus non/burst-SCS use cohort (Table 2). Acute bone- and muscle-related complications occurred in similar proportions in both cohorts. Patients in the SCS use cohort also more frequently had hypertension, diabetes, and cardiac arrhythmias compared with those in the non/burst-SCS use cohort (Additional File 1; Supplementary Table 3).

### SCS exposure

The mean length of post-index follow-up period was similar in the SCS use cohort (24.6 months) and the non/burst-SCS use cohort (24.2 months) (Table 1). The number of patients in both cohorts decreased with follow-up length. Of the 7473 patients in the SCS use cohort at index, 5818 (77.9%) were observed for ≥ 1 year post-index, 3257 (43.6%) for ≥ 2 years post-index, and 1684 (22.5%) for ≥ 3 years post-index (Additional File 1; Supplementary Table 4). During the first quarter of follow-up (Months 1–3), the SCS use cohort included 3175 (42.5%) patients with low SCS exposure, 2532 (33.9%) patients with medium SCS exposure, and 1766 (23.6%) patients with high SCS exposure, and 452 (6.0%) patients had continuous high exposure. These proportions remained similar throughout the follow-up period. The proportion of patients in the SCS use cohort with ≥ 3 and ≥ 4 SCS bursts was initially low (0.8% and 0.0%, respectively) and increased throughout the follow-up period to 66.7% and 56.4%, respectively, at Months 73–75.

### SCS-related complications during the follow-up period

**Fig. 2:**
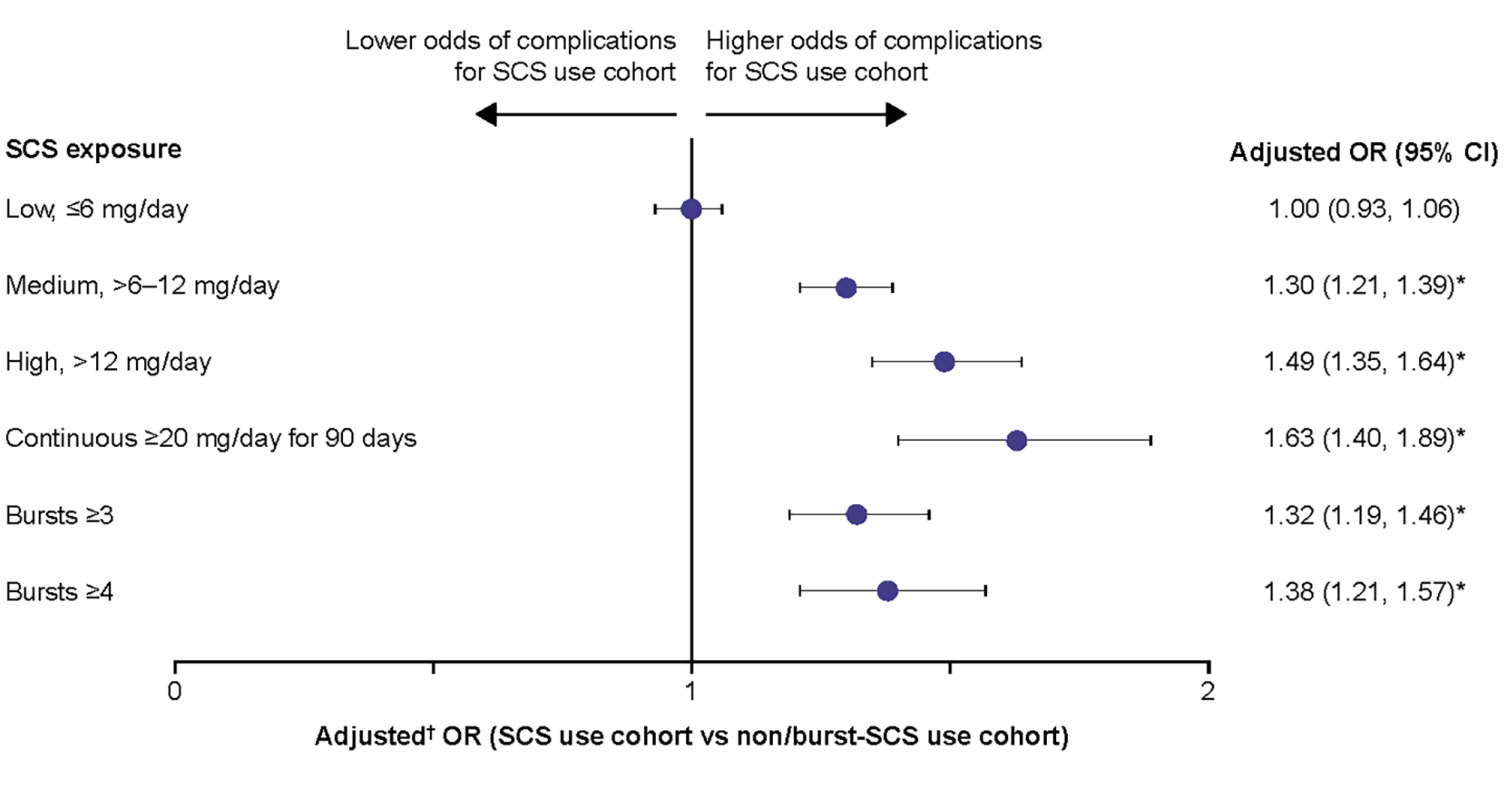
Odds of any SCS-related complications during the follow-up period due to SCS exposure. Prednisone-equivalent SCS dose; *p-value < 0.05; ^**†**^from multivariate GEE models adjusting for patient demographics and baseline clinical characteristics (see Methods for covariates). CI, confidence interval; GEE, generalized estimating equations; OR, odds ratio; SCS, systemic corticosteroid

Patients with medium, high, and continuous high SCS dose, along with patients with ≥ 3 and ≥ 4 SCS bursts, had significantly greater odds (adjusted OR: 1.30–1.63) of developing any SCS-related complication than the non/burst-SCS use cohort (Fig. 2). Numerically, the greatest odds for developing any SCS-related complication were in patients with continuous high SCS dose (Fig. 2).

**Fig. 3:**
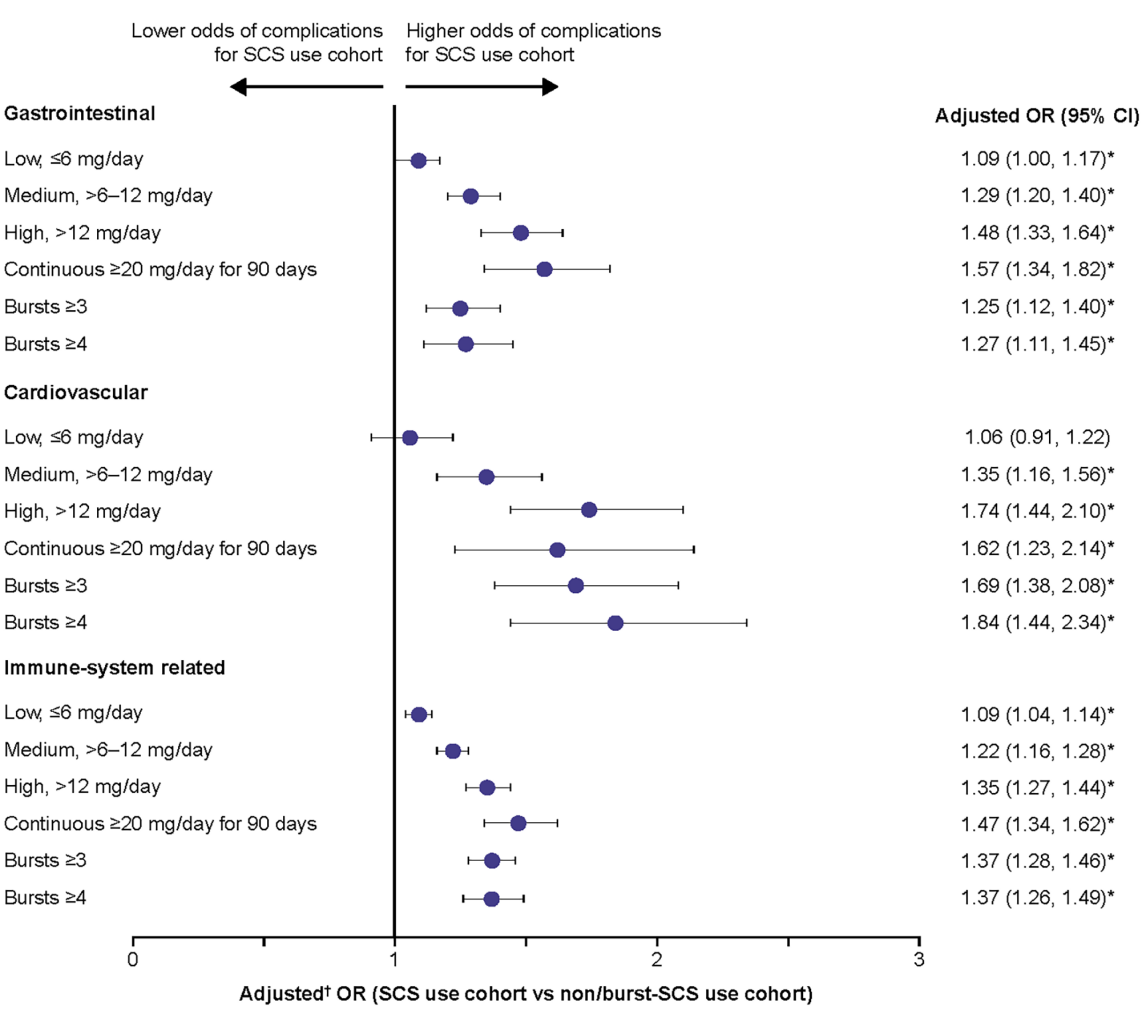
Odds of acute SCS-related complications during the follow-up period due to SCS exposure. Prednisone-equivalent SCS dose; *p-value < 0.05; ^**†**^from multivariate GEE models adjusting for patient demographics/baseline clinical characteristics (see Methods for covariates). CI, confidence interval; GEE, generalized estimating equations; OR, odds ratio; SCS, systemic corticosteroid; SD standard deviation

Patients with medium, high, and continuous high SCS dose, along with those with ≥ 3 and ≥ 4 SCS bursts, had significantly greater odds of developing acute gastrointestinal (adjusted OR: 1.25–1.57), cardiovascular (adjusted OR: 1.35–1.84), and immune system-related (adjusted OR: 1.22–1.47) complications than the non/burst-SCS use cohort (Fig. 3). Patients with low-dose SCS also had significantly greater odds of developing acute gastrointestinal (adjusted OR: 1.09) and immune system-related (adjusted OR: 1.09) complications than the non/burst-SCS use cohort (Fig. 3).

**Fig. 4:**
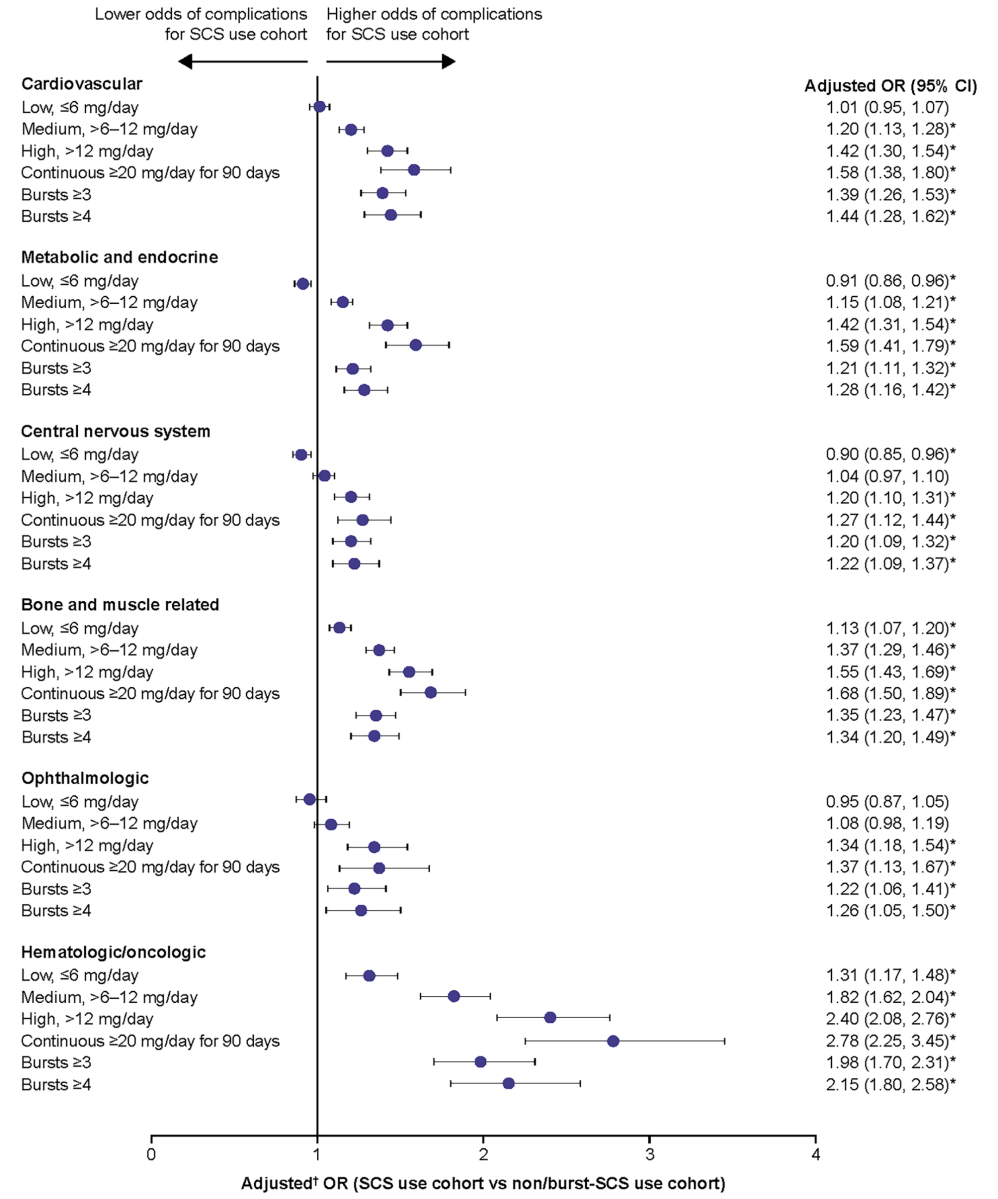
Odds of chronic SCS-related complications during the follow-up period due to SCS exposure. Prednisone-equivalent SCS dose; *p-value < 0.05; ^**†**^from multivariate GEE models adjusting for patient demographics and baseline clinical characteristics (see Methods for covariates). CI, confidence interval; GEE, generalized estimating equations; OR, odds ratio; SCS, systemic corticosteroid

For chronic SCS-related complications, patients with medium, high, and continuous high SCS dose, along with patients with ≥ 3 and ≥ 4 SCS bursts, had significantly higher odds of developing any of the complications assessed versus the non/burst-SCS use cohort, with the exception of central nervous system and ophthalmologic complications in the medium-dose subgroup (Fig. 4). In the low-dose subgroup, the odds of developing a bone and muscle-related or hematologic/oncologic complication were significantly increased versus the non/burst-SCS use cohort. The highest ORs were observed for hematologic/oncologic complications. The increased odds of chronic complications were dose-dependent, with the greatest odds for complications in patients with continuous high SCS dose. Overall proportions of patients with complications within each category are shown in Additional File 1, Supplementary Table 5. In the sensitivity analysis excluding patients with any SCS exposure during follow-up, patients with low SCS dose (≤ 6 mg/day) had increased odds of most SCS-related complications when compared with the non/burst-SCS user cohort (Additional File 2; Supplementary Fig. 2).

### HCRU due to SCS-related complications

**Fig. 5:**
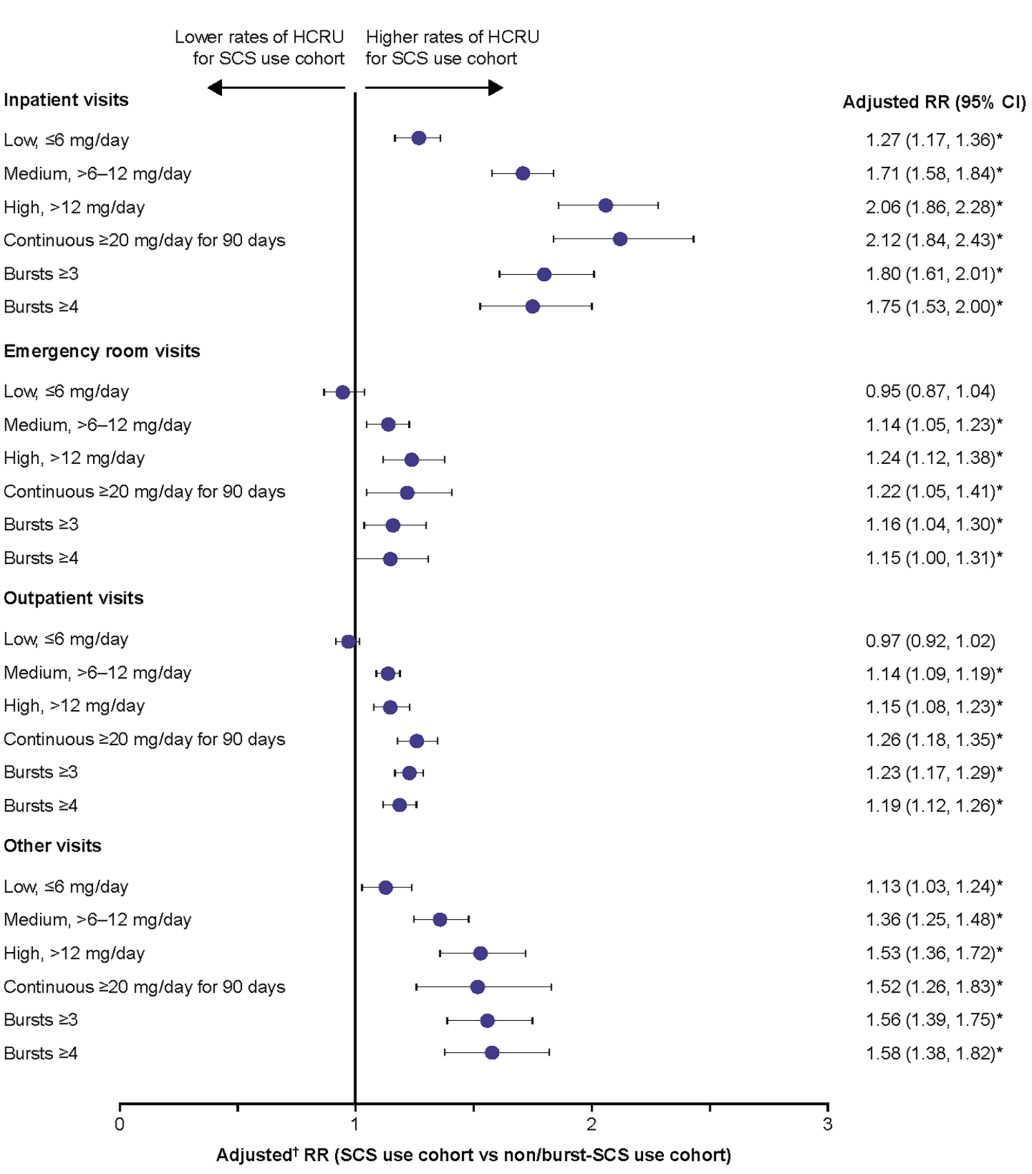
Rate of HCRU due to SCS-related complications during the follow-up period. Prednisone-equivalent SCS dose; *p-value < 0.05; ^†^from multivariate GEE models adjusting for patient demographics/baseline clinical characteristics (see Methods for covariates). CI, confidence interval; GEE, generalized estimating equations; HCRU, healthcare resource utilization; RR, rate ratio; SCS, systemic corticosteroid

The rates of all types of HCRU with a diagnosis code for SCS-related complications during the follow-up period were higher in patients with medium, high, and continuous high SCS dose, and in those with ≥ 3 and ≥ 4 SCS bursts, compared with the non/burst-SCS use cohort (adjusted rate ratio [RR]: 1.14–2.12) (Fig. 5). Patients with a low SCS dose had a statistically significantly higher rate of IP visits (adjusted RR: 1.27) and other visits (adjusted RR 1.13) than those in the non/burst-SCS use cohort, although the adjusted RR was lower than for patients with medium, high, and continuous high SCS dose.

### SCS treatment patterns in the SCS use cohort

In the SCS use cohort, the mean daily dose of SCS gradually decreased between Months 1–3 (9.4 mg/day) and Months 28–30 (to 6.5 mg/day), then stabilized at approximately 6–7 mg/day for the remainder of the follow-up period (Additional File 1; Supplementary Table 6).

## Discussion

This real-world retrospective study in patients with severe asthma using data from a large US claims database found that both burst and chronic SCS use, even at low (≤ 6 mg/day) doses, significantly increases the odds of developing SCS-related complications. Similarly, burst and chronic SCS use in this population was linked with significantly higher rates of HCRU due to SCS-related complications, with even low chronic SCS doses associated with more frequent IP and other HCRU visits. These data support existing evidence of the well-documented and wide-reaching adverse effects associated with SCS use by highlighting a dose-dependent increase in the risk of complications among patients with severe asthma who continue to be frequent users of SCS therapy [5–10, 29]. They also suggest that even low doses of SCS are associated with complications and that complications occur with both maintenance and burst SCS use. Further, this study provides updated insight into the association between SCS use with related complications and HCRU in the era of biologic therapy.

In patients with a medium SCS dose or higher, including those with continuous high SCS dose and those with ≥ 3 or ≥ 4 SCS bursts, the odds of developing any SCS-related complication were 1.3–1.6 times greater than in the non/burst SCS use cohort. Notably, the increase in the risk of developing SCS-related complications was dose-dependent, with the greatest increase observed for patients with continuous high SCS use. These results build on previous findings that demonstrated the increased risk of SCS-related complications with greater SCS use in patients with asthma [5–8]. For example, in a retrospective claims analysis conducted in the US between 1997 and 2013, patients with medium (> 6–12 mg/day) and high (> 12 mg/day) SCS exposure had significantly higher risks of several different SCS-related complications versus those with low (≤ 6 mg/day) exposure, with ORs by complication type ranging from 1.29 to 2.12 for medium-dose users and 1.23 to 1.96 for high-dose users [7]. A further analysis of this dataset found a significant dose-response relationship between SCS exposure and any SCS-related complication, with ORs versus non-users increasing from 2.03 in low-dose (≤ 6 mg/day) users to 3.64 in high-dose (> 12 mg/day) users [8]. Similarly, in another retrospective claims analysis conducted in the US between 2003 and 2014, the odds of developing SCS-related complications increased in a dose-dependent manner with ORs compared with non-users ranging from 2.50 in patients with low SCS exposure (< 5 mg/day) to 3.32 in patients with high exposure (> 10 mg/day) [6]. The odds of developing any SCS-related complication reported in our study were lower in comparison to the studies conducted in 1997–2013 and 2003–2014 [6, 8], which may reflect the ongoing shift from regular use of SCS within asthma management. Together these data highlight the significant association between SCS dose and SCS-related complications in the asthma population and support the guideline-recommended aim of minimizing SCS use in these patients [2]. 

In line with previous findings [6–8], the current study found dose-dependent increases in the risk of acute gastrointestinal, cardiovascular and immune-system related complications as well as chronic cardiovascular, metabolic and endocrine, central nervous system, bone- and muscle-related, ophthalmologic, and hematologic/oncologic complications. Notably, even patients with low SCS exposure had increased risk of developing acute gastrointestinal and immune-system related complications as well as chronic bone- and muscle-related and hematologic/oncologic complications compared with the non/burst SCS use cohort. These findings are largely consistent with those from a previous focused literature review observing an increased risk of these complications with low-dose SCS versus no exposure among patients with severe asthma [35]. For other complications with low-dose versus no SCS exposure, results have been more mixed, with two studies finding no increase in the risk of cardiovascular complications, and for metabolic and ophthalmologic events, one study each showing a positive association and no association; [35] this largely accords with the lack of a significantly increased risk for these complications with low-dose SCS use in the current study. However, when patients with any SCS exposure during the follow-up period were excluded from the non/burst-SCS use cohort in a sensitivity analysis, the odds of all except ophthalmologic SCS-related complications were increased in the low-dose SCS subgroup compared with the modified non/burst-SCS use cohort. Overall, these data indicate an increased risk of complications even among those with low SCS use.

In addition to the increased risk of SCS-related complications, this study found higher rates of HCRU associated with SCS-related complications among patients with medium-dose SCS use or greater, with RRs versus non/burst SCS users ranging between 1.14 and 2.12. While there was some indication that higher SCS exposure correlated with higher HCRU than lower exposure, the dose-response relationship was not as clear as for SCS-related complications. An association between increased SCS exposure and higher HCRU due to SCS-related complications has also been found in previous retrospective claim studies [6–8]. Two of these studies also found that, compared with no SCS use even low SCS doses were associated with an increased rate of IP and other HCRU visits, consistent with the current study [6, 8]. Interestingly, Dalal et al., also found that low doses of SCS were associated with an increased rate of OP and ER visits compared with no SCS use [6]. Together, these findings indicate that beyond SCS-related complications, even low doses of SCS are associated with higher HCRU, suggesting a need for alternative corticosteroid-sparing management strategies among patients with severe asthma.

In the current study, baseline data generally showed higher comorbidity and concomitant medication use in SCS users versus non/burst-SCS users as well as higher all-cause and asthma-related healthcare costs. The proportion of SCS users without any controller therapy use appeared high at nearly 30%, which may indicate that some patients in the current study were using SCS as their controller therapy. These results appeared to align with other studies in asthma which did not require patients to be taking controller therapy. For example, in one claims analysis of patients with non-allergic asthma, 21.5% received fixed-dose ICS/ long-acting β2-agonists (LABA) during a 12-month baseline period, which was lower than the 42.5% during a 6-month baseline period in the current study [36]. While we did not collect data on healthcare costs during the follow-up period, the baseline differences may indicate that follow-up costs would also have been higher in SCS users, although their relationship with SCS-related complications is unclear. Outside of this study, it has been shown that healthcare costs are higher in SCS versus non-SCS users, with the costs associated with SCS-related complications increasing with SCS dose [5–9]. When combined with the adverse effects associated with SCS use, the higher healthcare costs among SCS users may be a further supporting factor for the use of SCS-sparing medications, such as biologics, in patients with severe asthma [16, 19, 20, 22, 24]. Furthermore, the cost effectiveness of biologic treatments has been demonstrated in a number of studies, particularly when treatment is targeted to specific responder populations [27, 28, 37, 38]. 

There are several limitations to the current study. As with other claims-based analyses, the data used were collected for payment rather than research purposes and are subject to coding limitations and may contain data entry errors. In particular, the categorization of complications as acute or chronic was based on the presence of an associated diagnosis code, which is contingent upon the treating physician’s judgement. As noted previously, the same caveat applies to HCRU given that encounters with a diagnosis code indicative of an SCS-related complication could also be due to uncontrolled asthma. Also, the presence of a dispensed medication does not indicate that the medication was taken as prescribed, nor that it was taken on the date of dispensing. In addition, patients with SCS bursts only or no bursts were not evaluated separately in this analysis. It should also be noted that eligibility for the non/burst-SCS use cohort precluded continuous SCS exposure for ≥ 6 months (the same criteria that defined patients in the SCS cohort as having severe asthma); consequently, differences in asthma severity between cohorts may have potentially impacted outcome differences between cohorts. Next, as ICS was not included in the definition of SCS use, it is possible that corticosteroid exposure was underestimated. However, since the bioavailability of ICS is lower than that of SCS, the impact of this exclusion is expected to be small. Additionally, the claims database used in this analysis includes data from 2014, prior to approval of several biologics for severe asthma in the US [11, 15, 17, 18]. However, the vast majority of patients included in the study had index dates after 2015. Therefore, the inclusion of data from 2014 may not have had a substantial effect on the relevance of this data for understanding the impact of SCS use since the introduction of biologic therapies. Finally, results may not be generalizable beyond the commercial and Medicare insurance population used. Nonetheless, these data provide valuable information on the burden of SCS use in patients with severe asthma and support a reduction of their use wherever possible in this population.

## Conclusions

In conclusion, these findings add to the currently available evidence on the risks of SCS use for patients with severe asthma and characterize the continued impact of SCS use for healthcare providers, policy makers, and payers. As such, they provide further support for the use of SCS-sparing therapies such as biologics for the treatment of severe asthma.

### Electronic supplementary material

Below is the link to the electronic supplementary material.

Supplementary Material 1. Additional File 1: Supplementary Tables.docx

Supplementary Material 2. Additional File 2: Supplementary Figures.docx

## Data Availability

To access data for GSK sponsored research, please submit an enquiry via www.gsk-studyregister.com/en/.

## Abbreviations

CI: confidence interval
CCI: Charlson comorbidity index
CDM: Clinformatics Data Mart
CM: Clinical Modification
ER: emergency room
GEE: generalized estimating equations
GINA: Global Initiative for Asthma
HCRU: healthcare resource utilization
ICD: International Classification of Diseases
ICS: inhaled corticosteroid
IP: inpatient
LABA: long-acting β2-agonist
LAMA: long-acting muscarinic antagonist
OCS: oral corticosteroids
OP: outpatient
OR: odds,ratio
RR: rate ratio
SABA: short-acting β2-agonist
SAMA: short-acting muscarinic antagonist
SCS: systemic corticosteroid
SD: standard deviation
