# Supplementary material for: Real-world association between systemic corticosteroid exposure and complications in US patients with severe asthma
Source: Allergy Asthma Clin Immunol. 2024 Mar 26;20:25. doi: 10.1186/s13223-024-00882-y (PMC10964513; doi:10.1186/s13223-024-00882-y)
Supplement: Supplementary file 1 — Supplementary Material 1. Additional File 1: Supplementary Tables.docx [file 13223_2024_882_MOESM1_ESM.docx]

# Supplementary Figures

**Supplementary Figure 1.** Patient disposition

***
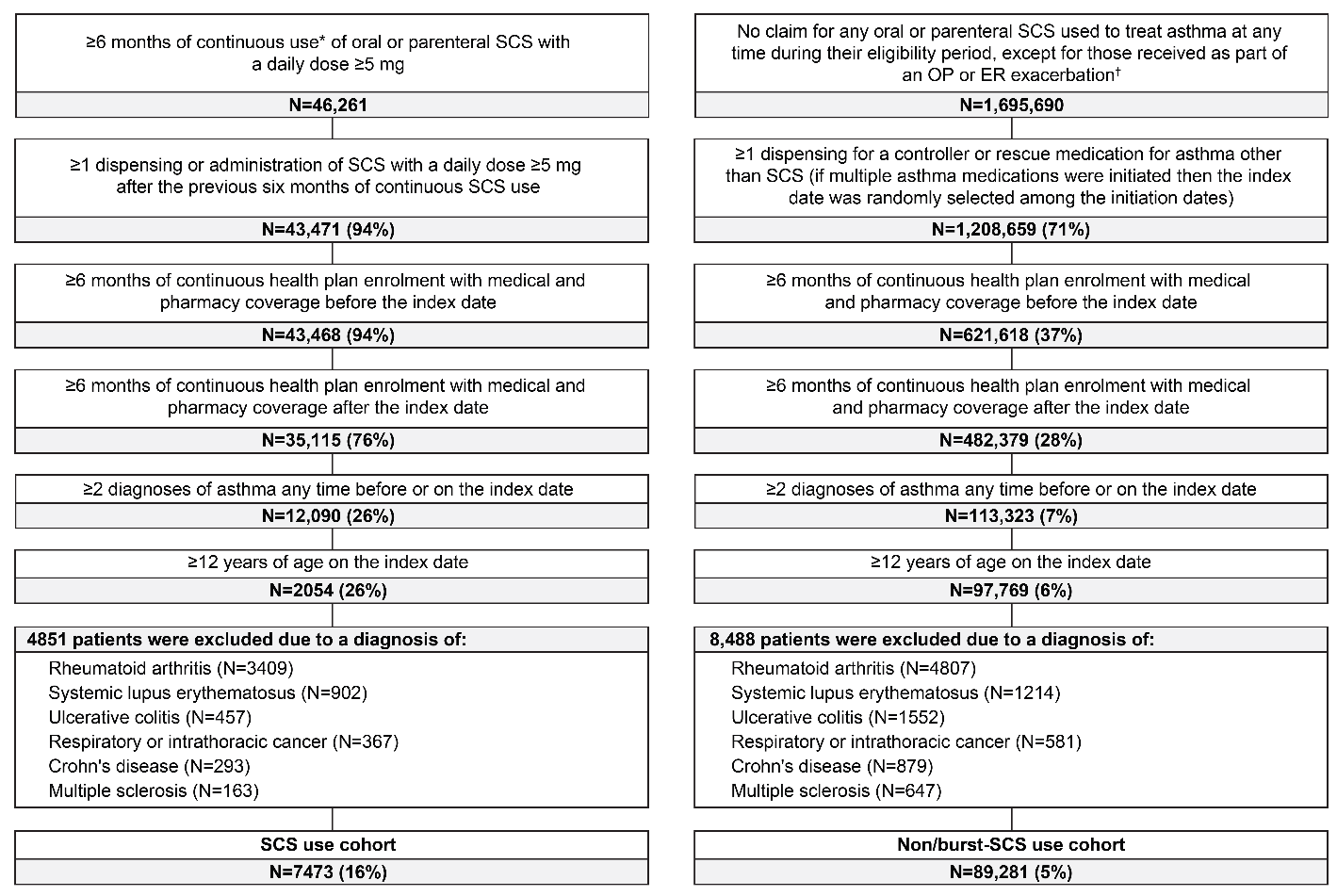
***

*No gap >14 days between claims; claims on the same day were considered to have been taken concurrently if the SCS agent was the same or consecutively if the SCS agent was different; ^†^defined as an asthma-related OP or ER visit with ≥1 claim for an SCS within -4/+5 days of the encounter.

ER, emergency room; OP, outpatient; SCS, systemic corticosteroid.

**Supplementary Figure 2**. Impact of low dose (≤6 mg/day) SCS exposure on SCS-related complications compared with non/burst-SCS use cohort after excluding patients with any SCS exposure during the follow-up period


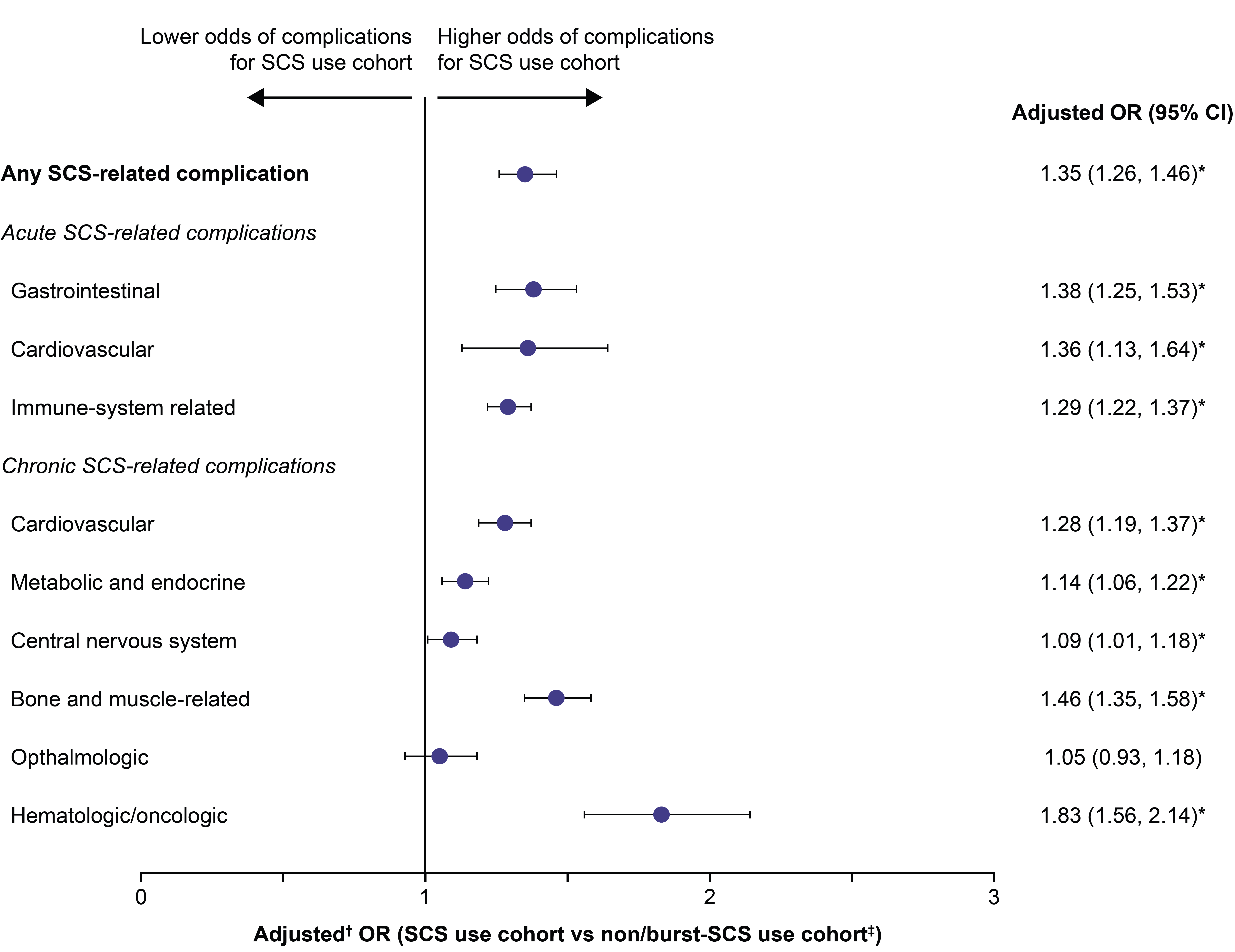


*p-value <0.05; ^†^from multivariate GEE models adjusting for patient demographics and baseline clinical characteristics (see Methods for covariates); ^‡^patients in the SCS non/burst-SCS user cohort with any SCS exposure during follow-up were excluded. The sample size decreased from N=89,281 to N=38,586.

CI, confidence interval; OR, odds ratio; SCS, systemic corticosteroid.
