# Supplementary material for: Real-world association between systemic corticosteroid exposure and complications in US patients with severe asthma
Source: Allergy Asthma Clin Immunol. 2024 Mar 26;20:25. doi: 10.1186/s13223-024-00882-y (PMC10964513; doi:10.1186/s13223-024-00882-y)
Supplement: Supplementary file 2 — Supplementary Material 2. Additional File 2: Supplementary Figures.docx [file 13223_2024_882_MOESM2_ESM.docx]

**Supplementary Tables**

**Supplementary Table 1**. ICD-9-CM and ICD-10-CM diagnosis codes for asthma and other conditions

| **Condition** | **Diagnosis codes** | |
| --- | --- | --- |
| Asthma | ICD-9-CM | 493.0x, 493.1x, 493.8x, 493.9x |
|  | ICD-10-CM | J45.3x, J45.4x, J45.5x, J45.9xx |
| Any cancer of the respiratory and intrathoracic system | ICD-9-CM | 160.x–165.x |
|  | ICD-10-CM | C30.x–C39.x |
| Rheumatoid arthritis | ICD-9-CM | 714.0x, 714.2x |
|  | ICD-10-CM | M06.x |
| Crohn’s disease | ICD-9-CM | 555.x |
|  | ICD-10-CM | K50.x |
| Ulcerative colitis | ICD-9-CM | 556.x |
|  | ICD-10-CM | K51.x |
| Systemic lupus erythematosus | ICD-9-CM | 710.0x |
|  | ICD-10-CM | M32.x |
| Multiple sclerosis | ICD-9-CM | 340.x |
|  | ICD-10-CM | G35.x |

CM, Clinical Modification; ICD, International Classification of Diseases.

**Supplementary Table 2**. SCS-related complications

| **Category** | **Conditions** |
| --- | --- |
| **Acute complications** |  |
| Gastrointestinal effects | Nausea and vomiting, gastrointestinal hemorrhage and ulcers, flatulence and related conditions, dyspepsia,  acute pancreatitis |
| Cardiovascular | Old myocardial infarction, acute myocardial infarction |
| Immune system-related | Pneumonia, fungal infection, urinary tract infection, tuberculosis, varicella infection, sepsis |
| Bone and muscle related | Bursitis |
| **Chronic complications** |  |
| Cardiovascular | Hypertension, heart failure, atrial fibrillation and flutter, stroke |
| Metabolic and endocrine | Hyperlipidemia, diabetes, obesity, Cushing syndrome, metabolic syndrome, hyperglycemia, drug-induced adrenocortical insufficiency |
| Central nervous system | Sleep disorders, anxiety disorders, depressive disorders, bipolar disorder, steroid psychosis, migraines, akathisia |
| Bone and muscle related | Back pain, muscle weakness, osteoporosis, fracture, avascular necrosis, loss of muscle mass, myopathy |
| Ophthalmologic | Glaucoma, cataract |
| Hematologic/oncologic | Elevated white blood cell count, epistaxis, malignant neoplasm of bladder |
| Dermatologic | Acne, erythema, hirsutism |
| Gastrointestinal | Chronic gastritis |

SCS, systemic corticosteroid.

**Supplementary Table 3**. Additional patient baseline demographics and clinical characteristics

| Characteristics | SCS use cohort  N=7473 | Non/burst-SCS use cohort  N=89,281 | Standardized difference* |
| --- | --- | --- | --- |
| Year of index date^†^, n (%) |  |  |  |
| 2014 | 165 (2.2) | 1667 (1.9) | 2.4 |
| 2015 | 320 (4.3) | 4791 (5.4) | 5.1 |
| 2016 | 1138 (15.2) | 13,483 (15.1) | 0.4 |
| 2017 | 1515 (20.3) | 18,245 (20.4) | 0.4 |
| 2018 | 1687 (22.6) | 19,155 (21.5) | 2.7 |
| 2019 | 1816 (24.3) | 20,144 (22.6) | 4.1 |
| 2020 | 832 (11.1) | 11,796 (13.2) | 6.4 |
| Elixhauser comorbidities^‡,§^, n (%) |  |  |  |
| Hypertension | 5181 (69.3) | 36,827 (41.2) | 56.5* |
| Uncomplicated | 3599 (48.2) | 30,048 (33.7) | 29.5* |
| Complicated | 1582 (21.2) | 6779 (7.6) | 38.7* |
| Diabetes | 2689 (36.0) | 17,430 (19.5) | 36.8* |
| Uncomplicated | 1743 (23.3) | 10,414 (11.7) | 30.7* |
| Complicated | 946 (12.7) | 7016 (7.9) | 15.8* |
| Cardiac arrhythmias | 2155 (28.8) | 10,883 (12.2) | 41.2* |
| Obesity | 1728 (23.1) | 15,702 (17.6) | 13.7* |
| Congestive heart failure | 1715 (22.9) | 6191 (6.9) | 44.9* |
| Hypothyroidism | 1597 (21.4) | 11,701 (13.1) | 21.9* |
| Fluid and electrolyte disorders | 1554 (20.8) | 6737 (7.5) | 38.0* |
| Renal failure | 1482 (19.8) | 6011 (6.7) | 38.6* |
| Peripheral vascular disorders | 1218 (16.3) | 5699 (6.4) | 31.3* |
| Rheumatoid arthritis/collagen vascular disease | 927 (12.4) | 1765 (2.0) | 40.4* |
| Valvular disease | 890 (11.9) | 5004 (5.6) | 22.3* |
| Pulmonary circulation disorder | 805 (10.8) | 2439 (2.7) | 32.0* |
| Deficiency anemias | 740 (9.9) | 3554 (4.0) | 23.3* |
| Solid tumor without metastasis | 679 (9.1) | 2999 (3.4) | 23.7* |
| Other neurological disorders | 557 (7.5) | 3222 (3.6) | 16.8* |
| Liver disease | 493 (6.6) | 3155 (3.5) | 14.0* |
| Coagulopathy | 428 (5.7) | 1678 (1.9) | 20.1* |
| Asthma-related comorbidities^§^, n (%) |  |  |  |
| Gastroesophageal reflux disease | 2553 (34.2) | 15,945 (17.9) | 37.2* |
| Obstructive sleep apnea | 1729 (23.1) | 9890 (11.1) | 32.0* |
| Depression | 1686 (22.6) | 14,336 (16.1) | 16.5* |
| Allergic rhinitis | 1566 (21.0) | 21,155 (23.7) | 6.6 |
| Sinusitis | 1182 (15.8) | 13,558 (15.2) | 1.7 |
| Other medications^§^, n (%) |  |  |  |
| Antihypertensives | 5054 (67.6) | 35,343 (39.6) | 56.2* |
| Antihyperlipidemics | 3410 (45.6) | 24,364 (27.3) | 38.1* |
| Proton pump inhibitors | 3318 (44.4) | 17,661 (19.8) | 52.7* |
| Antidiabetics | 2063 (27.6) | 13,182 (14.8) | 31.4* |
| Anticoagulants | 1490 (19.9) | 6590 (7.4) | 36.6* |
| Bisphosphonates | 601 (8.0) | 1567 (1.8) | 29.1* |
| *Psychiatric medications* |  |  |  |
| Antidepressants | 2866 (38.4) | 22,269 (24.9) | 28.8* |
| Anxiolytics | 2181 (29.2) | 13,251 (14.8) | 34.6* |
| Thyroid hormone | 1653 (22.1) | 11,313 (12.7) | 24.9* |
| Mood stabilizers/anticonvulsants | 1601 (21.4) | 9842 (11.0) | 28.2* |
| Atypical antipsychotics | 336 (4.5) | 2913 (3.3) | 6.4 |
| Psychostimulants | 117 (1.6) | 2665 (3.0) | 9.5 |
| Typical antipsychotics | 119 (1.6) | 472 (0.5) | 10.4* |
| Other antipsychotics | 1 (0.0) | 4 (0.0) | 0.9 |
| Healthcare costs^§,║^, USD 2020, mean (SD) |  |  |  |
| All-cause |  |  |  |
| *Total healthcare costs* | 35,714 (62,517) | 11,733 (28,744) | 49.3* |
| Total medical costs | 27,832 (56,450) | 10,077 (27,336) | 40.0* |
| IP visit costs | 10,927 (36,637) | 3,830 (19,525) | 24.2* |
| ER visit costs | 3,043 (10,488) | 1,785 (5,974) | 14.7* |
| OP visit costs | 12,197 (34,471) | 4,028 (13,056) | 31.3* |
| Other visit costs | 1,664 (6,417) | 435 (3,045) | 24.5* |
| Pharmacy costs | 7,882 (19,175) | 1,657 (6,855) | 43.2* |
| *Total patient-paid costs* |  |  |  |
| Medical costs | 1,086 (1,717) | 730 (1,394) | 22.7* |
| Pharmacy costs | 605 (1,000) | 248 (366) | 47.5* |
| Asthma-related |  |  |  |
| *Total healthcare costs* | 4,641 (17,802) | 1,882 (8,260) | 19.9* |
| Total medical costs | 2,790 (17,278) | 1,485 (8,137) | 9.7 |
| IP visit costs | 1,450 (15,572) | 705 (7,169) | 6.1 |
| ER visit costs | 336 (2,805) | 401 (2,099) | 2.6 |
| OP visit costs | 971 (5,358) | 366 (2,628) | 14.3* |
| Other visit costs | 33 (251) | 12 (222) | 8.7 |
| Pharmacy costs | 1,851 (3,354) | 397 (1,029) | 58.6* |
| *Total patient-paid costs* |  |  |  |
| Medical costs | 131 (537) | 129 (528) | 0.3 |
| Pharmacy costs | 199 (340) | 79 (156) | 45.2* |

*Standardized difference: ≥10%; ^†^evaluated on the index date; ^‡^present in ≥5% of patients; ^§^evaluated during the 6-month baseline period, excluding the index date; ^║^costs were inflation-adjusted to USD 2020 using the US Medical Care consumer price index.

ER, emergency room; IP, inpatient; OP, outpatient; SCS, systemic corticosteroid; SD, standard deviation; USD, United States dollar.

**Supplementary Table 4.** Description of SCS exposure per quarter of follow-up among SCS users

| **Follow-up period, months** | **Patients, N** | **Average daily dose, mg/day, mean ± SD [median]** | **SCS exposure cohort*, n (%)** | | | | | |
| --- | --- | --- | --- | --- | --- | --- | --- | --- |
|  |  |  | **Low**  **(≤6 mg/day)** | **Medium**  **(>6–12 mg/day)** | **High**  **(>12 mg/day)** | **Continuous high dose**^†^  **(≥20 mg/day)** | **Bursts**^‡^ **≥3** | **Bursts**^‡^ **≥4** |
| **1–3** | 7473 | 9.4 ± 9.5 [7] | 3175 (42.5) | 2532 (33.9) | 1766 (23.6) | 452 (6.0) | 60 (0.8) | 2 (0.0) |
| **4–6** | 7473 | 9.2 ± 9.5 [7] | 3084 (41.3) | 2715 (36.3) | 1674 (22.4) | 565 (7.6) | 360 (4.8) | 104 (1.4) |
| **7–9** | 6623 | 8.9 ± 8.2 [7] | 2741 (41.4) | 2499 (37.7) | 1383 (20.9) | 446 (6.7) | 651 (9.8) | 300 (4.5) |
| **10–12** | 5818 | 8.6 ± 7.7 [7] | 2509 (43.1) | 2197 (37.8) | 1112 (19.1) | 358 (6.2) | 827 (14.2) | 437 (7.5) |
| **13–15** | 5044 | 8.2 ± 7.4 [7] | 2280 (45.2) | 1855 (36.8) | 909 (18.0) | 267 (5.3) | 914 (18.1) | 540 (10.7) |
| **16–18** | 4322 | 8.2 ± 13.2 [7] | 1984 (45.9) | 1604 (37.1) | 734 (17.0) | 229 (5.3) | 920 (21.3) | 604 (14.0) |
| **19–21** | 3780 | 8.1 ± 16.9 [6] | 1782 (47.1) | 1363 (36.1) | 635 (16.8) | 194 (5.1) | 938 (24.8) | 625 (16.5) |
| **22–24** | 3257 | 8.2 ± 23.3 [6] | 1568 (48.1) | 1154 (35.4) | 535 (16.4) | 166 (5.1) | 927 (28.5) | 620 (19.0) |
| **25–27** | 2798 | 8.3 ± 28.7 [6] | 1344 (48.0) | 998 (35.7) | 456 (16.3) | 134 (4.8) | 889 (31.8) | 606 (21.7) |
| **28–30** | 2304 | 8.2 ± 28.5 [6] | 1120 (48.6) | 830 (36.0) | 354 (15.4) | 111 (4.8) | 781 (33.9) | 567 (24.6) |
| **31–33** | 1996 | 8.2 ± 27.8 [6] | 983 (49.2) | 715 (35.8) | 298 (14.9) | 86 (4.3) | 722 (36.2) | 541 (27.1) |
| **34–36** | 1684 | 7.5 ± 6.6 [6] | 831 (49.3) | 618 (36.7) | 235 (14.0) | 89 (5.3) | 664 (39.4) | 503 (29.9) |
| **37–39** | 1379 | 7.5 ± 6.6 [6] | 673 (48.8) | 508 (36.8) | 198 (14.4) | 67 (4.9) | 571 (41.4) | 433 (31.4) |
| **40–42** | 1089 | 7.6 ± 6.6 [6] | 541 (49.7) | 374 (34.3) | 174 (16.0) | 51 (4.7) | 488 (44.8) | 387 (35.5) |
| **43–45** | 894 | 7.5 ± 6.3 [6] | 443 (49.6) | 309 (34.6) | 142 (15.9) | 37 (4.1) | 416 (46.5) | 337 (37.7) |
| **46–48** | 705 | 7.8 ± 6.3 [6] | 335 (47.5) | 244 (34.6) | 126 (17.9) | 40 (5.7) | 351 (49.8) | 278 (39.4) |
| **49–51** | 540 | 8.0 ± 6.6 [7] | 250 (46.3) | 188 (34.8) | 102 (18.9) | 25 (4.6) | 286 (53.0) | 230 (42.6) |
| **52–54** | 365 | 8.0 ± 6.8 [7] | 164 (44.9) | 130 (35.6) | 71 (19.5) | 18 (4.9) | 213 (58.4) | 172 (47.1) |
| **55–57** | 260 | 8.0 ± 7.2 [7] | 115 (44.2) | 96 (36.9) | 49 (18.8) | 8 (3.1) | 165 (63.5) | 140 (53.8) |
| **58–60** | 187 | 8.5 ± 7.7 [7] | 78 (41.7) | 74 (39.6) | 35 (18.7) | 11 (5.9) | 121 (64.7) | 105 (56.1) |
| **61–63** | 134 | 8.7 ± 8.4 [7] | 58 (43.3) | 49 (36.6) | 27 (20.1) | 6 (4.5) | 90 (67.2) | 79 (59.0) |
| **64–66** | 106 | 9.0 ± 8.9 [7] | 46 (43.4) | 36 (34.0) | 24 (22.6) | 5 (4.7) | 74 (69.8) | 67 (63.2) |
| **67–69** | 81 | 9.6 ± 9.8 [7] | 36 (44.4) | 26 (32.1) | 19 (23.5) | 3 (3.7) | 57 (70.4) | 53 (65.4) |
| **70–72** | 55 | 10.2 ± 10.9 [7] | 23 (41.8) | 18 (32.7) | 14 (25.5) | 4 (7.3) | 38 (69.1) | 34 (61.8) |
| **73–75** | 39 | 8.2 ± 6.1 [6] | 17 (43.6) | 14 (35.9) | 8 (20.5) | 3 (7.7) | 26 (66.7) | 22 (56.4) |

*SCS exposure was evaluated starting on the index date and updated for each quarter of follow-up, except for the continuous cohort, which was calculated for each quarter separately. Only complete quarters were evaluated. SCS exposure during the last quarter of follow-up (Months 76– 78) is not reported since it is not included in the regression models; ^†^defined as average daily dose ≥20 mg/day over 90 days with no gap >14 days; ^‡^defined as a pharmacy claim for SCS with 2–28 days of supply and an average daily dose equivalent to ≥20 mg prednisone (note that medical claims for SCS have 1 day of supply). Multiple SCS bursts less than 14 days apart were considered as one SCS burst.

SCS, systemic corticosteroid; SD, standard deviation.

**Supplementary Table 5.** Most frequently occurring SCS-related conditions during the follow-up period, per category listed

| **SCS-related complication categories** | **Patients with ≥1 complication during follow-up, n (%)** | |
| --- | --- | --- |
|  | **SCS use cohort N=7473** | **SCS non/burst-use cohort  N=89,281** |
| **Acute SCS-related complications, n (%)** |  |  |
| *Gastrointestinal* |  |  |
| Nausea and vomiting | 1998 (26.7) | 14,728 (16.5) |
| Gastrointestinal hemorrhage | 709 (9.5) | 3529 (4.0) |
| Flatulence and related conditions | 670 (9.0) | 4115 (4.6) |
| *Cardiovascular* |  |  |
| Old myocardial infarction | 748 (10.0) | 3188 (3.6) |
| Acute myocardial infarction | 564 (7.5) | 2062 (2.3) |
| *Immune system related* |  |  |
| Pneumonia | 3527 (47.2) | 21,339 (23.9) |
| Fungal infection | 2374 (31.8) | 14,996 (16.8) |
| Urinary tract infection | 2160 (28.9) | 13,977 (15.7) |
| **Chronic SCS-related complications, n (%)** |  |  |
| *Cardiovascular* |  |  |
| Hypertension | 6109 (81.7) | 45,785 (51.3) |
| Heart failure | 2646 (35.4) | 9583 (10.7) |
| Atrial fibrillation and flutter | 1860 (24.9) | 7545 (8.5) |
| *Metabolic and endocrine* |  |  |
| Hyperlipidemia | 4503 (60.3) | 33,661 (37.7) |
| Diabetes | 3454 (46.2) | 22,341 (25.0) |
| Obesity | 3367 (45.1) | 34,196 (38.3) |
| *Central nervous system* |  |  |
| Sleep disorders | 3491 (46.7) | 26,404 (29.6) |
| Anxiety disorders | 2930 (39.2) | 25,345 (28.4) |
| Depressive disorders | 2736 (36.6) | 21,726 (24.3) |
| *Bone and muscle related* |  |  |
| Back pain | 3372 (45.1) | 26,006 (29.1) |
| Muscle weakness | 1895 (25.4) | 7745 (8.7) |
| Osteoporosis | 1893 (25.3) | 7280 (8.2) |
| Fracture | 1519 (20.3) | 8181 (9.2) |
| *Ophthalmologic* |  |  |
| Glaucoma | 1257 (16.8) | 8532 (9.6) |
| Cataract | 961 (12.9) | 5155 (5.8) |
| *Hematologic/oncologic* |  |  |
| Elevated white blood cell count | 1591 (21.3) | 5425 (6.1) |
| Epistaxis | 269 (3.6) | 1581 (1.8) |
| Malignant neoplasm of bladder | 72 (1.0) | 296 (0.3) |

SCS, systemic corticosteroid.

**Supplementary Table 6.** SCS treatment patterns among the SCS use cohort during follow-up

| **Follow-up period, months** | **Patients,  N** | **Number of SCS claims mean (SD) [median]** | **Total dose^*^, mg,  mean (SD) [median]** | **Daily dose, mg, mean^†^ (SD) [median]** | **Cumulative total dose^‡^, mg, mean (SD) [median]** | **Cumulative daily dose^§^, mg, mean (SD) [median]** | **Patients with ≥1 SCS burst**^║^**, n (%)** |
| --- | --- | --- | --- | --- | --- | --- | --- |
| **1–3** | 7473 | 2.8 (2.0) | 847 (852) [655] | 9.4 (9.5) [7] | 847 (852) [655] | 9.4 (9.5) [7] | 1931 (25.8) |
| **4–6** | 7473 | 2.4 (1.9) [2] | 813 (1,066) [621] | 9.0 (11.8) [7] | 1660 (1702) [1320] | 9.2 (9.5) [7] | 1532 (20.5) |
| **7–9** | 6623 | 2.2 (2.0) [2] | 748 (834) [550] | 8.3 (9.3) [6] | 2393 (2213) [1934] | 8.9 (8.2) [7] | 1357 (20.5) |
| **10–12** | 5818 | 2.0 (2.0) [2] | 685 (738) [476] | 7.6 (8.2) [5] | 3080 (2768) [2502] | 8.6 (7.7) [7] | 1122 (19.3) |
| **13–15** | 5044 | 1.9 (1.9) [1] | 648 (722) [450] | 7.2 (8.0) [5] | 3707 (3311) [3005] | 8.2 (7.4) [7] | 974 (19.3) |
| **16–18** | 4322 | 1.9 (2.0) [1] | 718 (6,120) [450] | 8.0 (68.0) [5] | 4408 (7106) [3513] | 8.2 (13.2) [7] | 854 (19.8) |
| **19–21** | 3780 | 1.8 (1.9) [1] | 669 (3,331) [450] | 7.4 (37.0) [5] | 5122 (10,618) [3995] | 8.1 (16.9) [6] | 679 (18.0) |
| **22–24** | 3257 | 1.8 (1.8) [1] | 714 (5,663) [450] | 7.9 (62.9) [5] | 5891 (16,763) [4491] | 8.2 (23.3) [6] | 609 (18.7) |
| **25–27** | 2798 | 1.7 (1.8) [1] | 693 (5,334) [450] | 7.7 (59.3) [5] | 6724 (23,234) [5103] | 8.3 (28.7) [6] | 522 (18.7) |
| **28–30** | 2304 | 1.7 (1.8) [1] | 581 (794) [445] | 6.5 (8.8) [5] | 7391 (25,628) [5518] | 8.2 (28.5) [6] | 422 (18.3) |
| **31–33** | 1996 | 1.7 (1.9) [1] | 575 (801) [440] | 6.4 (8.9) [5] | 8108 (27,571) [6038] | 8.2 (27.8) [6] | 358 (17.9) |
| **34–36** | 1684 | 1.7 (2.1) [1] | 569 (713) [448] | 6.3 (7.9) [5] | 8106 (7133) [6591] | 7.5 (6.6) [6] | 310 (18.4) |
| **37–39** | 1379 | 1.6 (1.9) [1] | 555 (688) [430] | 6.2 (7.6) [5] | 8775 (7729) [7105] | 7.5 (6.6) [6] | 222 (16.1) |
| **40–42** | 1089 | 1.7 (1.9) [1] | 553 (664) [445] | 6.1 (7.4) [5] | 9544 (8308) [7610] | 7.6 (6.6) [6] | 213 (19.6) |
| **43–45** | 894 | 1.7 (1.8) [1] | 553 (630) [445] | 6.1 (7.0) [5] | 10,156 (8558) [8209] | 7.5 (6.3) [6] | 185 (20.7) |
| **46–48** | 705 | 1.7 (1.8) [1] | 571 (677) [450] | 6.3 (7.5) [5] | 11,171 (9119) [9150] | 7.8 (6.3) [6] | 126 (17.9) |
| **49–51** | 540 | 1.7 (1.9) [1] | 595 (889) [445] | 6.6 (9.9) [5] | 12,241 (10,138) [10,204] | 8.0 (6.6) [7] | 93 (17.2) |
| **52–54** | 365 | 1.8 (2.2) [1] | 625 (1,046) [450] | 6.9 (11.6) [5] | 12,980 (11,069) [10,920] | 8.0 (6.8) [7] | 73 (20.0) |
| **55–57** | 260 | 1.7 (1.7) [1] | 593 (933) [400] | 6.6 (10.4) [4] | 13,734 (12,335) [11,496] | 8.0 (7.2) [7] | 66 (25.4) |
| **58–60** | 187 | 1.5 (1.7) [1] | 574 (714) [420] | 6.4 (7.9) [5] | 15,276 (13,911) [12,445] | 8.5 (7.7) [7] | 28 (15.0) |
| **63–63** | 134 | 1.7 (1.9) [1] | 570 (731) [450] | 6.3 (8.1) [5] | 16,480 (15,968) [12,901] | 8.7 (8.4) [7] | 32 (23.9) |
| **64–66** | 106 | 1.7 (2.1) [1] | 567 (783) [409] | 6.3 (8.7) [5] | 17,828 (17,666) [13,278] | 9.0 (8.9) [7] | 16 (15.1) |
| **67–69** | 81 | 1.6 (1.5) [1] | 636 (983) [401] | 7.1 (10.9) [4] | 19,786 (20,274) [13,955] | 9.6 (9.8) [7] | 16 (19.8) |
| **70–72** | 55 | 1.6 (1.9) [1] | 693 (953) [450] | 7.7 (10.6) [5] | 22,073 (23,553) [14,500] | 10.2 (10.9) [7] | 10 (18.2) |
| **73–75** | 39 | 1.7 (2.2) [1] | 548 (644) [400] | 6.1 (7.2) [4] | 18,430 (13,673) [14,528] | 8.2 (6.1) [6] | 4 (10.3) |
| **76–78** | 14 | 1.6 (2.1) [1] | 326 (368) [160] | 3.6 (4.1) [2] | 17,355 (7828) [14,863] | 7.4 (3.3) [6] | 2 (14.3) |
| **Follow-up^**^** |  |  |  |  |  |  |  |
| PPPQ | 7473 | 2.1 (1.4) [2] | 707 (1610) [552] | 8.4 (18.4) [7] | - | - | 4338 (58.0) |

Prednisone-equivalent SCS dose.

*Total dose per quarter is given by the product of dose per day and days of supply in the corresponding period; ^†^average daily dose per quarter is given by the total dose divided by 90 days. Average daily dose for the entire follow-up is given by the average total dose over the entire follow-up divided by the number of complete quarters in days (i.e., 90 days per complete quarter); ^‡^cumulative total dose during follow-up is given by the cumulative total dose since index divided by the number of days between the index date and the end of the corresponding quarter; ^§^cumulative average daily dose during follow-up is given by the cumulative total dose since index divided by the number of days between the index date and the end of the corresponding quarter; ^║^SCS bursts were defined as a pharmacy claim for SCS with 2–28 days of supply and an average daily dose equivalent to prednisone ≥20 mg (note that medical claims for SCS have 1 day of supply). Multiple SCS bursts less than 14 days apart were considered as one SCS burst; **entire follow-up values are calculated for each patient over their entire follow-up period divided by the duration of the follow-up period in complete quarters. Patients are weighted by their observation period in complete quarters.

PPPQ, per patient per quarter; SCS, systemic corticosteroid; SD, standard deviation.
